# Isolation of *Spirosoma foliorum* sp. nov. from the fallen leaf of *Acer palmatum* by a novel cultivation technique

**DOI:** 10.1038/s41598-023-35108-5

**Published:** 2023-09-06

**Authors:** Ho Le Han, Dian Alfian Nurcahyanto, Neak Muhammad, Yong-Jae Lee, Tra T. H. Nguyen, Song-Gun Kim, Sook Sin Chan, Kuan Shiong Khoo, Kit Wayne Chew, Pau Loke Show, Thi Ngoc Thu Tran, Thi Dong Phuong Nguyen, Chen Yaw Chiu

**Affiliations:** 1 The University of Danang, University of Science and Technology, 54 Nguyen Luong Bang St., Danang, 550 000 Viet Nam; 2 Research Center for Biosystematics and Evolution, Research Organization for Life Sciences and Environment, National Research and Innovation Agency (BRIN), Cibinong 16911, West Java, Indonesia; 3 Biological Resource Center/Korean Collection for Type Cultures (KCTC), Korea Research Institute of Bioscience and Biotechnology, 181 Ipsingil, Jeongeup, 56212 Jeonbuk Korea; 4 University of Science and Technology (UST), 217 Gajeong-Ro, Yuseong, Daejeon, 34113 Korea; 5 Institut Sains Biologi, Fakulti Sains, Universiti Malaya, Kuala Lumpur, Malaysia; 6 Department of Chemical Engineering and Materials Science, Yuan Ze University, Taoyuan, Taiwan; 7 Centre for Herbal Pharmacology and Environmental Sustainability, Chettinad Hospital and Research Institute, Chettinad Academy of Research and Education, Kelambakkam, 603103 Tamil Nadu India; 8 School of Chemistry, Chemical Engineering and Biotechnology, Nanyang Technological University, 62 Nanyang Drive, Singapore, 637459 Singapore; 9 Department of Chemical Engineering, Khalifa University, Shakhbout Bin Sultan St - Zone 1, Abu Dhabi, United Arab Emirates; 10 Department of Chemical and Environmental Engineering, Faculty of Science and Engineering, University of Nottingham Malaysia, Jalan Broga, 43500 Semenyih, Selangor Darul Ehsan Malaysia; 11 The University of Da Nang, University of Technology and Education, Da Nang City, 550000 Viet Nam; 12 Biochemical Engineering Research Center, Ming Chi University of Technology, New Taipei City, 24301 Taiwan

**Keywords:** Bacterial evolution, Bacterial genetics

## Abstract

In the effort of isolating novel microbial species, the strain PL0132^T^ was isolated from a fallen leaf under fresh water at a stream, which glided when grown on a tap water medium (without nutrients). The strain was determined to be Gram-negative, strictly aerobic, and rod-shaped, which grew optimally at 25 °C, pH 6–7, and the strain tolerates 1% (w/v) NaCl concentration. The complete genome of strain PL0132^T^ comprises one contig with a sequencing depth of 76×, consisting of 8,853,064 base pairs and the genomic DNA G + C content was 46.7% (genome). 16S rRNA gene sequence analysis revealed that strain PL0132^T^ represents a member of the phylum *Bacteroidetes* and is affiliated with the genus *Spirosoma*. Based on genomic, phenotypic, and chemotaxonomic characteristics, the strain PL0132^T^ represents a novel species of the genus *Spirosoma*, for which the name *Spirosoma foliorum* sp. nov. is proposed (= KCTC 72228^ T^ = InaCC B1447^T^).

## Introduction

Microbial diversity on the earth is affluent, and 99% of microbial species are still unknown^1^. The limitation of the traditional culture method is one of the drawbacks of isolating novel microbial species and few microorganisms have been cultivated by current techniques^2^. Therefore, new methods for microbial isolation and cultivation should be invested in and studied more.

The genus *Spirosoma* belongs to the family *Cytophagaceae* which is the largest family belonging to the phylum *Bacteroidetes*^3^. The genus *Spirosoma* was originally described by Larkin and Borrall in 1984^4^ and was emended by Finster et al.^5^ and Ahn et al.^6^ At the time of writing, the genus *Spirosoma* comprised 44 valid names (https://lpsn.dsmz.de/genus/spirosoma), including 12 recently described species: *Spirosoma taeanense* TS118^T^^7^, *Spirosoma endbachense* -24^T^^8^, *Spirosoma telluris* HMF3257^T^, *Spirosoma arboris* HMF4905^T^^9^, *Spirosoma agri* S7-3-3^T^^10^, *Spirosoma terrae* 15J9-4^T^^11^, *Spirosoma jeollabukense* S2-3-6^T^^12^, *Spirosoma pollinicola* HA7^T^^13^, *Spirosoma humi* S7-4-1^T^^14^, *Spirosoma horti* S7-3-19^T^^15^, *Spirosoma harenae* 15J8-9^T^^16^ and *Spirosoma pomorum* S7-2-11^T^^17^.

The species of the genus *Spirosoma* have been isolated from soil, dust, air, water, and extreme conditions like Arctic glaciers^18–20^. Furthermore, the characteristics of the genus include Gram-stain-negative, strictly aerobic, non-spore-forming, yellow or orange pigmented bacteria which are characterized as menaquinone MK-7 as the respiratory quinone, phosphatidylethanolamine as the major polar lipid, and summed feature 3 (C_16: 1ω6C_ and/or C_16: 1ω7C_) C_16:1ω5c_, iso-C_15:0_, and C_16:0_ as the major fatty acids^6^. In this paper, a gliding bacterium was isolated from a decaying leaf of *Acer palmatum* in a stream of fresh water. The distinctive characteristics led us to propose a novel species in the genus *Spirosoma*.

## Results and discussion

**Figure 1:**
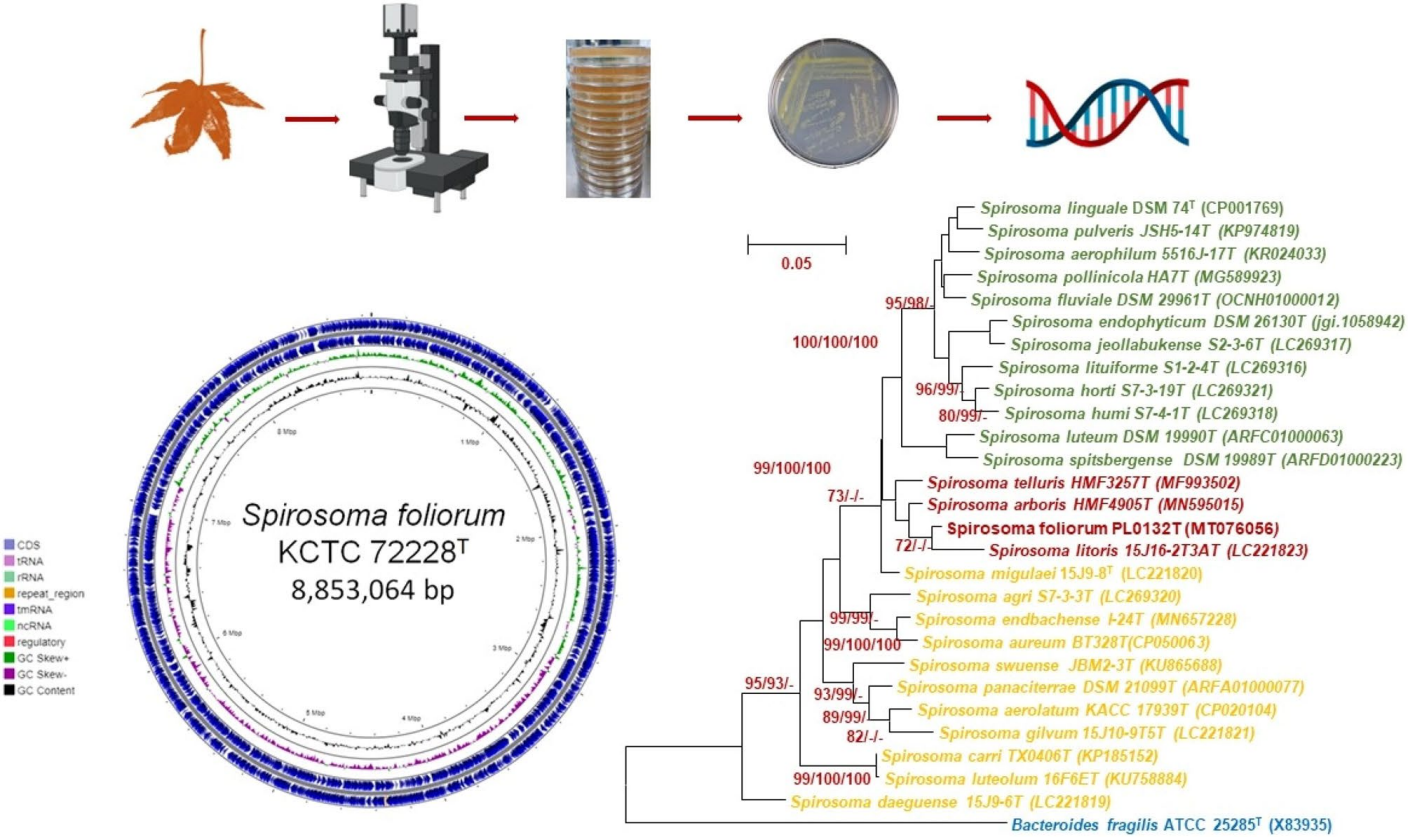
Maximum-likelihood phylogenetic tree, based on 16S rRNA gene sequences, showing the phylogenetic position of *Spirosoma foliorum* PL0132^T^ among related strains of the genus *Spirosoma*. Closed circles indicate that the corresponding nodes were also recovered in the tree generated with the neighbor-joining and maximum parsimony algorithm. Bootstrap values in the order ML/NJ/MP are indicated as percentages of 1000 replications datasets, when greater than 70%. The tree was rooted using *Bacteroides fragilis* ATCC 25285^T^ (X83935) as an outgroup. Bar, 0.05 substitutions per nucleotide position.

For phylogenetic characterisation, the comparative 16S rRNA gene sequence results revealed that the strain was phylogenetically affiliated with the genus *Spirosoma*. The 16S rRNA gene of PL0132^T^ showed similarity values of 97.9%, 97.1%, 96.4% and 96.4% to *Spirosoma arboris* HMF4905^T^, *Spirosoma litoris* 15J16-2T3A^T^, *Spirosoma migulaei* 15J9-8^T^ and *Spirosoma telluris* HMF3257^T^, respectively (Figs. 1, S1 and S2).

The complete genome sequence of strain PL0132^T^ comprised one contig with a sequencing depth of 76×; the contig was 8,853,064 nt long with a circular structure. The GC contents of the chromosome were 46.7%. Gene annotation by NCBI Prokaryotic Genome Annotation Pipeline identified 7,408 genes which include 7,126 genes coding protein, 9 rRNAs (5S, 3; 16S, 3; 23S, 3), and 44 tRNAs. The complete genome was deposited in the GenBank/EMBL/DDBJ under the accession number CP059732. The identification of secondary metabolite biosynthesis gene cluster was determined by using antiSMASH^21^. Secondary metabolite clusters annotated by antiSMASH included terpene synthase genes, polyketide synthase type I and III (T1PKS and T3PKS), unspecified ribosomally synthesized and post-translationally modified peptide product cluster (RiPP-like), RRE-element containing cluster and non-ribosomal peptide synthetase (NRPS) (Fig. S5).

The average nucleotide identity (ANI) scores between the genomic sequence of PL0132^T^ and *Spirosoma arboris* HMF4905^T^, *Spirosoma migulaei* 15J9-8^ T^, and *Spirosoma telluris* HMF3257^T^ were 80.81, 79.13 and 79.72%^9^, respectively, which were determined by the ANI Calculator of EzBiocloud service (www.ezbiocloud.net/tools/ani). Digital DNA-DNA hybridization (dDDH) values were also calculated using the Genome Blast Distance Phylogeny version 2.1 web browser from DSMZ (http://ggdc.dsmz.de). The dDDH values between the whole genome sequences of strain PL0132^T^ and its closest relatives were 24.40, 22.7, and 23.30% for *Spirosoma arboris* HMF4905^T^, *Spirosoma migulaei* 15J9-8^T^, and *Spirosoma telluris* HMF3257^T^, respectively. These values of ANI and dDDH were below the standard cut-off criteria for ANI (95–96%)^22^ and dDDH (70%)^23^, indicating that strain PL0132^T^ represents a novel species of the genus *Spirosoma*.

**Figure 2:**
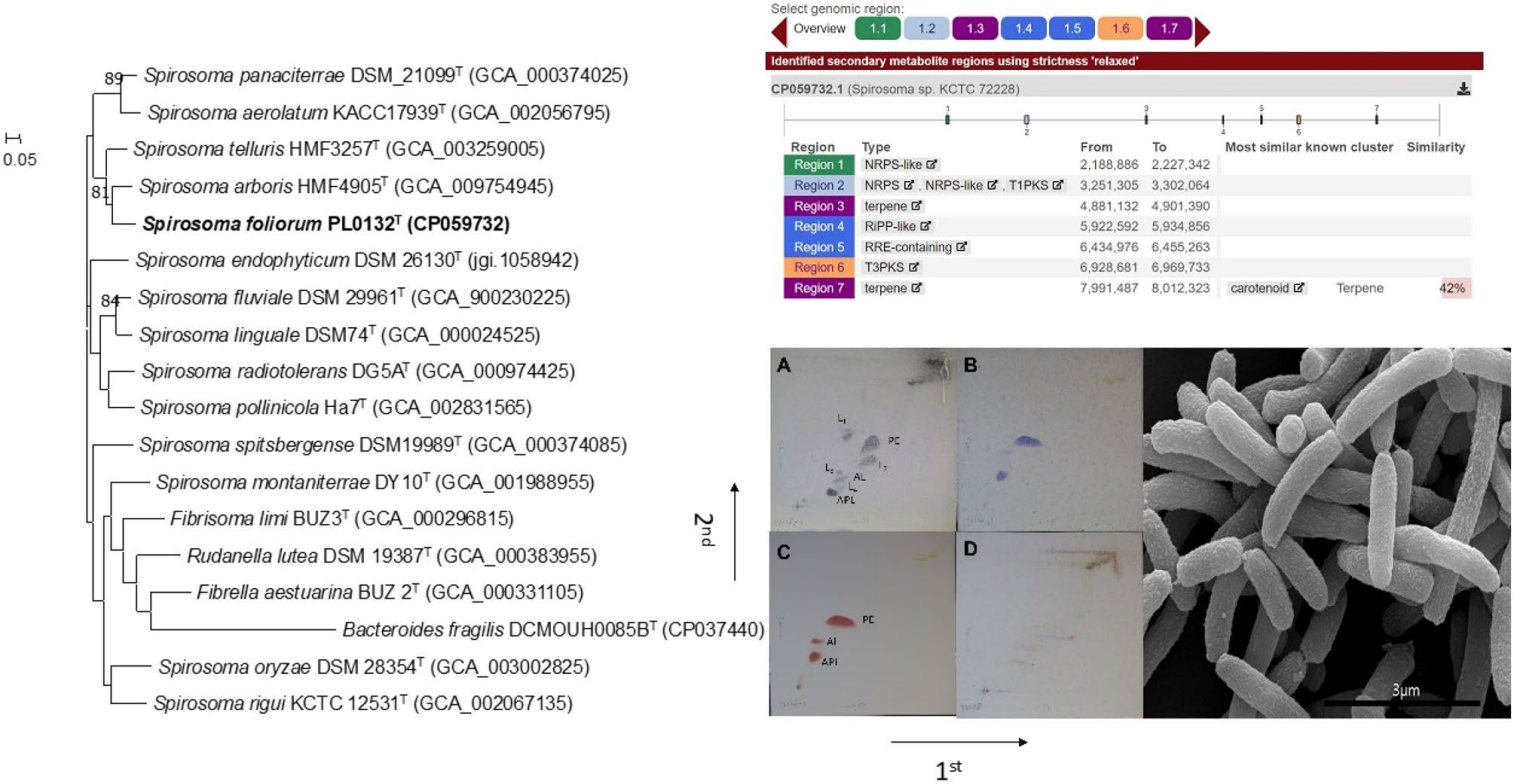
Genome-based phylogenetic tree of strain *Spirosoma foliorum* PL0132^T^ and other related type strains using UBCGs (concatenated alignment of 92 core genes). Bootstrap values are indicated at nodes. Scale bar, 0.05 substitutions per position; Predicted gene clusters of secondary metabolites biosynthesis annotated in antiSMASH against strain PL0132^T^ complete genome. Analyses provided the identification of six clusters involved in biosynthesis of terpene synthase genes, polyketide synthase type I and III (T1PKS and T3PKS), unspecified ribosomally synthesized and post-translationally modified peptide product cluster (RiPP-like), RRE-element containing cluster and non-ribosomal peptide synthetase (NRPS); Two-dimensional TLC patterns of the total polar lipids of strain *Spirosoma foliorum* PL0132^T^ with The following spray reagents were used for detection: (**A**) molybdatophosphoric acid (for total lipids); (**B**) molybdenum blue (for phospholipids); (**C**) ninhydrin (for amino lipids); (**D**) α-naphthol (for glycolipids). Phosphatidylethanolamine (PE), Amino lipid (AL), Amino Phospho Lipid (APL), and unidentified lipids (L1-L4).1st: first dimension; 2nd: second dimension; Scanning electron micrographs (SEM) of strain *Spirosoma foliorum* PL0132^T^ grown on R2A for 2 days at 25 °C. Bars 3 μm.

To determine the taxonomic position of strain PL0132^T^, a genome-based phylogenetic tree was reconstructed using an up-to-date bacterial core gene (UBCG) set consisting of 92 genes, as described by Na et al.^24^ Briefly, the core genes identified (hmmsearch; v3.1b2) from predicted CDSs (Prodigal; v2.6.3) were concatenated, and aligned (MAFFT; v7.310), and subjected to construct a UBCG tree. The phylogenomic tree showed that PL0132^T^ formed a clade with *S. arboris* HMF4905^T^ within the genus *Spirosoma* (Fig. 2).

The strain PL0132^T^ was Gram-stain-negative, strictly aerobic, non-spore-forming, catalase and oxidase-positive, rod-shaped without flagella, approximately 0.8–1.0 μm wide, 2–6 μm long (Fig. S3). The strain glided when grown on a tap water medium (without nutrients). Colonies grown on R2A for 48 h were circular, convex, and pale yellow. Growth occurred at 4–30 °C (optimum 25 °C), at pH 6.5–8.5 (optimum 7), and the strain did not require NaCl for its growth, but it tolerated at a concentration up to 1% (w/v) NaCl. The isolated strain grew on R2A and NA, and weakly on TSA but did not grow on LB agar. Physiological and biochemical characteristics of the isolated strain were described in the species description. Phenotypic and chemotaxonomic properties that differentiated strain PL0132^T^ from its closest relatives in the genus are listed in Table 1.

**Table 1:** Differential phenotypic characteristics of *Spirosoma foliorum* PL0132^T^ and phylogenetically closely related *Spirosoma* species.

Characteristic	1	2	3	4	5
Growth at/on					
10 °C	+	−	−	−	+
Nutrient agar	−	+	+	−	+
Tryptic soy agar	+	−	−	−	+
Catalase activity	+	+	−	+	+
Enzyme activity (API ZYM)					
Esterase (C4)	+	+	+	−	+
α-Galactosidase	+	−	+	−	+
β-Glucosidase	+	−	−	−	−
α-Mannosidase	+	−	+	+	−
Assimilation of L-arabinose (API 20NE)	−	+	−	+	−
Assimilation of potassium gluconate	−	−	−	+	−
Acid production from (API 50CH)					
Amidon (starch)	−	−	−	−	+
Amygdalin	+	+	−	+	+
D-Arabinose	−	+	+	+	+
L-Arabinose	+	+	+	+	−
Arbutin	+	+	−	+	+
Gentiobiose	+	−	−	−	+
Inulin	+	−	−	−	+
D-Lyxose	+	+	+	−	+
D-Melezitose	+	−	−	+	+
D-Raffinose	+	−	+	+	+

Strains: (1) PL0132^T^; (2) *S. arboris* KCTC 72779^ T^; (3) *S. litoris* KCTC 52029^ T^; (4) *S. telluris* KCTC 62463^ T^; (5) *S. migulaei* KCTC 52028^ T^.All strains were positive for oxidase activity, hydrolysis of aesculin; leucine arylamidase, valine acrylamidase, acid phosphatase, naphthol-AS-BI-phosphohydrolase, β-galactosidase, α-glucosidase activities; assimilation of D-glucose, D-mannose, and D-maltose; production of acid from aesculin, arbutin, N-acetylglucosamin, cellobiose, D-fructose, D-galactose, D-glucose, D-lactose, D-mannose, D-maltose, melibiose, methyl α-D-mannopyranoside, methyl α-D-glucopyranoside, saccharose, salicin, trehalose and D-xylose. All strains were negative for nitrate reduction, indole production, glucose fermentation, urease, gelatinase, lipase (C14), β-glucuronidase, α-fucosidase activities; assimilation of adipic acid, capric acid, D-mannitol, malic acid, trisodium citrate, and phenylacetic acid; production of acid from D-adonitol, ducitol, glycogen, glycerol, inositol, D-manitol, methyl β-D-xylopyranoside, L-sorbose, D-sorbitol, xylitol, potassium gluconate, and potassium 2-ketogluconate. + Positive; − Negative. All data were obtained in this study.

In the chemotaxonomic analysis, the fatty acids of strain PL0132^T^ were summed feature 3 as (C_16: 1_ ω6C and/or C_16: 1_ ω7C) (34.8%), C_16:1_ ω5c (18.6%), iso-C_15:0_ (20.0%), C_16:0_ (8.1%) and iso-C_17:0_ 3-OH (5.6%) (Table 2). The overall fatty acids of strain were similar to those of other *Spirosoma* species with the major components such as summed feature 3 and C_16:1_ ω5c. However, there were differences in the percentages of some components, particularly iso-C_15:0_ and C_16:0_.

**Table 2:** Cellular fatty acid profiles of strain PL0132^T^ and the type strains of related species of the genus *Spirosoma.*

Fatty acids	1	2	3	4	5
Straight-chain					
C_14:0_	1.1	0.7	0.9	2.4	0.6
C_16:0_	3.6	2.6	2.9	11.6	2.5
Branched					
Iso C_13:0_	–	–	–	0.6	0.4
Iso C_15:0_	11.4	15	12.0	4.1	12.1
Anteiso C_15:0_	–	1.6	–	0.9	1.2
Hydroxy					
Iso C_15:0_ 3-OH	2.4	2.1	2.3	3.5	2.4
Iso C_16:0_ 3-OH	–	1.9	–	–	–
C_16:0_ 3-OH	2.4	1.9	2.1	6.4	1.4
Iso C_17:0_	–	–	–	–	0.5
Iso C_17:0_ 3-OH	5.4	7.8	5.0	1.7	7.5
Unsaturated					
**C **_**16:1**_** ɷ5c**	**28.1**	**28.8**	**30.2**	**20.6**	**26.8**
Summed features*					
**3**	**45.6**	**39.6**	**44.7**	**46.9**	**43.2**
9	–	–	–	–	1.2

Strains: (1) PL0132^T^; (2) *S. arboris* KCTC 72779^T^; (3) *S. litoris* KCTC 52029^T^; (4) *S. telluris* KCTC 62463^T^; (5) *S. migulaei* KCTC 52028^T^. All data were obtained in this study. Values are percentages of the total fatty acids present, and only fatty acids accounting for more than 0.5% of the total. Major compositions are listed with bold numbers.

Summed feature 3 was listed as C_16:1ω6C_ and/or C_16:1ω7C_.

Summed feature 9 was listed as iso- C_17:1ω9C_ and/or 10-methyl C_16: 0._

The quinone of strain PL0132^T^ was menaquinone-7, which is similar to in other members of the genus *Spirosoma*^5, 6^. The polar lipid profile of PL0132^T^ showed the major lipids phosphatidylethanolamine (PE), two aminophospholipids (APL1-2), and four unidentified lipids (L1–L4) (Fig. S4). The profile was similar to other strains in the genus with major polar lipids, and the profile of the novel isolate strain had more unidentified lipids and minor amounts of aminophospholipid.

Strain PL0132^T^ had biochemical and physiological characteristics that differentiated strain PL0132^T^ from the other species of the genus *Spirosoma*. One difference was that *Spirosoma foliorum* PL0132^T^ and *S. migulaei* KCTC 52028^T^ could grow at 10 °C and on tryptic soy agar while the other closely species of genus *Spirosoma* do not. Second, *S.foliorum* PL0132^T^ and *S. migulaei* KCTC 52028^T^ could produce acid from gentiobiose and inulin while the type strains of *S. arboris*, *S. litoris*, and *S. telluris* could not (Table 1), however, *S.foliorum* PL0132^T^ could not produce acid from D-arabinose while the other close relative species could produce acid from D-arabinose. Third, it is important to note that glycolipid was found in other closely related species^9^ but not in *S. foliorum* PL0132^T^.

## Materials and methods

All experiments were carried out in accordance with relevant institutional, national, and international guidelines and legislation.

### Sampling sites and isolation

The strain PL0132^T^ was isolated from a fallen leaf of *Acer palmatum* under fresh water at a stream in Naejang mountain, in Jeongeup city, South Korea (35°28′48.3′′N 126°53′21.6′′E) in July 2018. The leaf was cut into a small piece of 5 × 5 mm and placed on a tap water agar medium without nutrients and incubated at 20 °C. After gliding motility was observed by a stereoscopic microscope, one strain was purified by transferring on Reasoner’s 2A (R2A) (BD Difco) agar. Finally, a bacterium with yellow color, designated PL0132^T^, was collected. Then, the strain was preserved in 15% (v/v) glycerol suspension at − 80 °C.

### 16S rRNA gene phylogeny

The genomic DNA of the strain PL0132^T^ was extracted from cells grown on R2A at 25 °C for 48 h. The 16S rRNA gene was amplified with two primers 27F and 1492R^25^. The sequence analysis was carried out by Sanger’s sequencing with an ABI3730XL automated sequencer (Applied Biosystems, USA). Then, the 16S rRNA gene sequences were uploaded to the EzBiocloud server^26^ to collect sequence information. All sequences of corresponding species obtained from the EzBiocloud were aligned and edited by BioEdit^27^ and CLUSTAL X software^28^. The phylogenetic trees were reconstructed by using neighbor-joining (NJ)^29^, maximum-likelihood (ML)^30^, and maximum parsimony (MP)^31^ methods in the MEGA 7 program^32^ with 1000 bootstrap replications.

### Genome sequencing and annotation

Whole-genome sequence of strain PL0132^T^ was determined by Nanopore technology. High molecular weight DNA was prepared and libraries were constructed following the native barcoding genomic DNA protocol (with EXP-NBD104, and SGK-LSK109, version NBE_9065_v109_revV_14Aug2019), after which sequencing was carried out. The library was loaded onto a MinION flow cell model FLO-MIN 106 (version R10.3). Then, sequencing was performed on MinKNOW platform (Oxford Nanopore Technologies). The resulting raw reads were quality assessed using pycoQC (2.5.2)^33^, and preprocessing was performed with porechop (0.2.4) to obtain 2.4 GB of reads. These reads were then processed using a custom pipeline based on Canu 2.0^34^, which was improved by referring to the CCBGpipe (Consensus Circular Bacterial Genome pipeline)^35^. Sequencing reads were produced through base calling on ONT Guppy software (version 3.2.10, Oxford Nanopore Technologies, Ltd., Oxford, UK), for 4 h. Assembling was done for reads which had good quality of quality scores ≥ 7 and sequence length ≥ 3000 bp. Before filtering, the mean read length was about 3,420 bp, and the read count was 675,410. After filtering, the mean read length was about 16,821 bp and the read count was 39,426. Subsequently, The assembled contigs were corrected and polishing was performed with Medaka^36^ (version 1.3.2, bacteria_odb10 1.3.2, https://github.com/nanoporetech/medaka) to improve genome quality and the completeness of the Nanopore assembly was evaluated and the quality of the assembled genome was confirmed with BUSCO using BUSCO^37^ (version 5.1.2, (https://busco.ezlab.org/, score: 94.4) and CheckM (version 1.1.3, score: 99.7)^38^, and annotations were added using PROKKA (1.1.2)^39^.

### Morphological and phenotypic analyses

Gram-staining was determined with a BD Gram-staining kit. Cells of the strain PL0132^T^ incubated 48 h on R2A (Difco) at 25 °C were used to examine the morphology using a FEI Quanta 250 FEG scanning electron microscope (FEI). Gliding motility was tested by microscopic hanging drop^16^. The phenotypic features were determined as follows: the optimal temperature for growth was investigated at 4, 10, 20, 25, 30, 37, and 45 °C on R2A agar after incubation for 7 days. Salt tolerance was examined using R2A broth containing 0, 0.5, 1, 2, 3, 4, 5 and 10% (w/v) NaCl. Growth at pH 5 to 10 (at intervals of 0.5 unit) was determined in R2A broth adjusted with various buffers, the concentration of 100 mM, acetate for pH 5–6, phosphate for pH 6.5–8, Tris for pH 8.5–9, and carbonate for pH 9.5–10^9^. Growth in the broth media was observed using OD_600_. Catalase and oxidase activity were carried out as described by Han et al.^40^ Growth on various media was assessed on R2A agar (Difco), Luria–Bertani agar (LB; Difco), nutrient agar (NA; Difco), and trypticase soy agar (TSA; Difco) after incubation for 7 days, at 25 °C. Anaerobic growth was determined by cultivation inside an anaerobic chamber (5% CO_2_, 5% H_2,_ and 90% N_2_) on R2A supplemented with 10 mM KNO_3_ at 25 °C for 7 days^41^. The hydrolysis of macromolecular compounds was determined on 1:10 strength R2A supplemented with skimmed milk (3%, w/v), starch (1%, w/v), dextrin (1%, w/v), and carboxymethyl-cellulose (1%, w/v) at 25°C^9^. Other enzyme activities and carbon source utilization ability were determined by using API 20NE, API 50CH, and API ZYM kits (bioMérieux).

### Chemotaxonomic analyses

To determine the fatty acid composition, cells of strain PL0132^T^ and the reference strains were collected from R2A agar at the same physiological age, by using the method of Sasser^42^. Fatty acid analyses were analyzed by the Sherlock Microbial Identification System (TSBA; library version 6.0)^43^. The isolated strain was cultured in R2A broth (Difco, BD) for 48 h to collect cell mass for isoprenoid quinone and polar lipid analysis. The isoprenoid quinone was extracted according to the method of Komagata and Suzuki^44^ from 100 mg freeze-dried cells using the solution of chloroform/methanol (2:1, v/v). The crude compound was purified by using a preparative TLC (20 mm × 20 mm, silica gel 60 F254 plates, Merck) with petroleum benzene/diethyl ether (9:1, v/v); then the compound was analyzed by using reverse-phase HPLC with a mixture of methanol and isopropyl ether (3:1, v/v), and detected by a UV detector. Polar lipids were extracted by a chloroform/methanol/water system, and then they were developed and separated using two-dimensional TLC^45^. The polar lipids were identified as described by Han et al.^46^.

## Conclusion

Based on the data for phylogenetic analysis, phenotypic and chemotaxonomic characteristics, PL0132^T^ represents a novel species of the genus *Spirosoma*. However, several phenotypic differences between the isolated strain and its phylogenetically related strains were summarized in Table 1. Therefore, PL0132^T^ should be classified as a novel species of the genus *Spirosoma*, for which the name *Spirosoma foliorum* sp. nov. is proposed.

## Description of *Spirosoma foliorum* sp. nov

*Spirosoma foliorum* sp. nov. (fo.li.o'rum. L. pl. gen. n. *foliorum*, of leaves, referring to the isolation of the type of strain from decaying leaves). The novel strain designated PL0132^T^ was isolated from decaying leaves under fresh water at a stream in Naejang mountain, in Jeongeup city, Republic of Korea.

Cells are Gram-stain-negative, and strictly aerobic rods, 0.8–1.0 μm wide, and 2–6 μm long. The strain glides when grown on a tap water medium (no nutrient). Colonies are convex, translucent, circular, and pale yellow. Growth occurs at occurred 4–30 °C (optimum 25 °C), at pH 6.5–8.5 (optimum 7), and the strain did not require NaCl for its growth. Tolerates 1% but not 2% (w/v) NaCl. Catalase and oxidase are positive. Starch, casein (skimmed milk), CM-cellulose, and DNA are not hydrolyzed. In API 20 NE tests, positive for aesculin hydrolysis, and β-galactosidase (PNPG test), but negative for gelatin hydrolysis, nitrate reduction, indole production, urease, and arginine dihydrolase. In assimilation of API 20NE, N-acetyl-D-glucosamine, D-glucose, D-maltose, and D-mannose are utilized, but adipate, L-arabinose, capric acid, potassium gluconate, malic acid, D-mannitol, trisodium citrate, and phenylacetate, are not utilized. In API ZYM tests, positive for acid phosphatase, alkaline phosphatase, *N*-acetyl-*β*-glucosaminidase, *ß*-glucuronidase, crystine arylamidase, esterase (C4), esterase (C8), *α*-galactosidase, *ß*-galactosidase (w), *α*-glucosidase (w), *ß*-glucosidase, leucine arylamidase, α-mannosidase, naphthol-AS-BI-phosphohydrolase, and valine arylamidase, but negative for *α*-chymotrypsin, *α*-fucosidase, trypsin and lipase (C14). In API 50 CH tests, acid is produced from *N*-acetyl-glucosamine, amygdalin, L-arabinose, arbutin, cellobiose, aesculin, D-fructose, D-galactose, gentiobiose, D-glucose, inulin, 5-ketogluconate, lactose, D-lyxose, D-mannose, D-maltose, melezitose, melibiose, methyl *α*-D-glucopyranoside, methyl *α*-D-mannopyranoside, raffinose, sucrose, salicin, trehalose, turanose, D-xylose but not from D-adonitol, D-arabinose, D-arabitol, L-arabitol, erythritol, D-fucose, L-fucose, gluconate, glycerol, glycogen, inositol, 2-ketogluconate, D-mannitol, methyl- *ß* -D-xylopyranoside, L-rhamnose, D-ribose, D-sorbitol, L-sorbose, starch, D-tagatose, xylitol, or L-xylose. The major cellular fatty acids of strain PL0132^T^ are summed feature 3 as C_16: 1ω6C_ and/or C_16: 1ω7C_ (45.6%), C_16:1ω5c_ (28.1%), iso-C_15:0_ (11.4%), C_16:0_ (3.6%) and iso-C_17:0_ 3-OH (5.4%). The only respiratory quinone of PL0132^T^ is MK-7. Phosphatidylethanolamine, aminolipid, aminophospholipid, and four unidentified lipids are the major polar lipid of PL0132^T^. The DNA G + C content is 46.7% (determined from the whole genome sequencing).

The type of strain, PL0132^T^ (= KCTC 72228^T^ = InaCC B1447^T^), was isolated from decayed leaves in Naejang mountain, Jeongeup city, Republic of Korea. The complete genome sequence of *Spirosoma foliorum* PL0132^T^ comprised one contig with a sequencing depth of 76×; the contig was 8,853,064 nt long with a circular structure. The GC contents of the chromosome were 46.7%.

### Supplementary Information

Supplementary Information.

## Data Availability

The 16S rRNA gene sequence and whole genome of strain *Spirosoma foliorum* PL0132^T^ generated and analysed during the current study are deposited the US National Institution of Health Genetic Sequence Database (GenBank), https://www.ncbi.nlm.nih.gov/genbank/; European Molecular Biology Laboratory (EMBL), https://www.ebi.ac.uk/; and the DNA Data Bank of Japan (DDBJ), http://getentry.ddbj.nig.ac.jp/getentry/na/MT076056/?filetype=html under accessions MT076056 and CP059732, respectively.

## Abbreviations

ANI: Average nucleotide identity
GGDC: Genome-to-genome distance calculator
SEM: Scanning electron microscopy
TLC: Thin-layer chromatography

## References

[1] Locey KJ, Lennon JT (2016). Scaling laws predict global microbial diversity. PNAS.

[2] Hahn, M. W., Koll, U. & Schmidt, J. *The Structure and Function of Aquatic Microbial Communities* (Springer International Publishing, 2019).

[3] Rosenberg E, DeLong EF, Lory S, Stackebrandt E, Thompson F (2014). The Prokaryotes: Actinobacteria.

[4] Larkin, J. M. & Borrall, R. *Family I. Spirosomaceae Larkin and Borrall 1978 595*^*AL*^*. *In* Bergey’s Manual of Systematic Bacteriology*. Vol. 1 ( Baltimore: Williams & Wilkins, 1984).

[5] Finster KW, Herbert RA, Lomstein BA (2009). *Spirosoma spitsbergense* sp. Nov. and *Spirosoma luteum* sp. Nov., isolated from a high Arctic permafrost soil, and emended description of the genus *Spirosoma*. Int. J. Syst. Evol. Microbiol..

[6] Ahn JH (2014). *Spirosoma oryzae* sp. nov., isolated from rice soil and emended description of the genus Spirosoma. Int. J. Syst. Evol. Microbiol..

[7] Lee JH (2021). *Spirosoma taeanense* sp. nov., a radiation resistant bacterium isolated from a coastal sand dune. Antonie Van Leeuwenhoek.

[8] Rojas J (2021). *Spirosoma endbachense* sp. nov., isolated from a natural salt meadow. Int. J. Syst. Evol. Microbiol..

[9] Kang H, Cha I, Kim H, Joh K (2020). *Spirosoma telluris* sp. nov. and *Spirosoma arboris* sp. nov. isolated from soil and tree bark, respectively. Int. J. Syst. Evol. Microbiol..

[10] Li W, Lee SY, Kang IK, Ten LN, Jung HY (2018). *Spirosoma agri* sp. nov., isolated from apple orchard soil. Curr. Microbiol..

[11] Ten LN (2018). *Spirosoma terrae* sp. nov., isolated from soil from Jeju Island, Korea. Curr. Microbiol..

[12] Li W, Ten LN, Lee SY, Lee DH, Jung HY (2018). *Spirosoma jeollabukense* sp. nov., isolated from soil. Arch. Microbiol..

[13] Ambika Manirajan B (2018). *Spirosoma pollinicola* sp. nov., isolated from pollen of common hazel (*Corylus avellana* L.). Int. J. Syst. Evol. Microbiol..

[14] Weilan L (2018). *Spirosoma humi* sp. nov., isolated from soil in South Korea. Curr. Microbiol..

[15] Li W, Ten LN, Lee SY, Kang IK, Jung HY (2018). *Spirosoma horti* sp. nov., isolated from apple orchard soil. Int. J. Syst. Evol. Microbiol..

[16] Ten LN (2018). *Spirosoma harenae* sp. nov., a bacterium isolated from a Sandy Beach. Curr. Microbiol..

[17] Li W, Lee SY, Kang IK, Ten LN, Jung HY (2018). *Spirosoma pomorum* sp. Nov., isolated from apple orchard soil. J. Microbiol..

[18] Okiria J (2017). *Spirosoma litoris* sp. nov., a bacterium isolated from beach soil. Int. J. Syst. Evol. Microbiol..

[19] Lee J-J (2017). *Spirosoma luteolum* sp. nov. isolated from water. J. Microbiol..

[20] Okiria J (2017). *Spirosoma migulaei* sp. nov., isolated from soil. J. Microbiol..

[21] Blin K (2021). antiSMASH 6.0: Improving cluster detection and comparison capabilities. Nucl. Acids Res..

[22] Yoon SH, Ha SM, Lim J, Kwon S, Chun J (2017). A large-scale evaluation of algorithms to calculate average nucleotide identity. Antonie Van Leeuwenhoek.

[23] Meier-Kolthoff JP, Auch AF, Klenk H-P, Göker M (2013). Genome sequence-based species delimitation with confidence intervals and improved distance functions. BMC Bioinf..

[24] Na SI (2018). UBCG: Up-to-date bacterial core gene set and pipeline for phylogenomic tree reconstruction. J Microbiol.

[25] Pheng S, Han HL, Park D-S, Chung CH, Kim S-G (2020). *Lactococcus kimchii* sp. nov., a new lactic acid bacterium isolated from kimchi. Int. J. Syst. Evol. Microbiol..

[26] Yoon SH (2017). Introducing EzBioCloud: A taxonomically united database of 16S rRNA gene sequences and whole-genome assemblies. Int. J. Syst. Evol. Microbiol..

[27] Hall TA (1999). BioEdit: A user-friendly biological sequence alignment editor and analysis program for Windows 95/98/NT. Nucl. Acids Symp. Ser..

[28] Thompson JD, Gibson TJ, Plewniak F, Jeanmougin F, Higgins DG (1997). The CLUSTAL_X windows interface: Flexible strategies for multiple sequence alignment aided by quality analysis tools. Nucl. Acids Res..

[29] Saitou N, Nei M (1987). The neighbor-joining method: A new method for reconstructing phylogenetic trees. Mol. Biol. Evol..

[30] Felsenstein J (1981). Evolutionary trees from DNA sequences: A maximum likelihood approach. J. Mol. Evol..

[31] Fitch WM (1971). Toward defining the course of evolution: Minimum change for a specific tree topology. Syst. Zool..

[32] Kumar S, Stecher G, Tamura K (2016). MEGA7: Molecular evolutionary genetics analysis version 7.0 for bigger datasets. Mol. Biol. Evol..

[33] Leonardi A, Leonardi A (2019). pycoQC, interactive quality control for Oxford Nanopore Sequencing. JOSS.

[34] Koren S (2017). Canu: Scalable and accurate long-read assembly via adaptive k-mer weighting and repeat separation. Genome Res..

[35] Liao YC (2019). Completing circular bacterial genomes with assembly complexity by using a sampling strategy from a single MinION run with barcoding. Front. Microbiol..

[36] Lee JY (2021). Comparative evaluation of Nanopore polishing tools for microbial genome assembly and polishing strategies for downstream analysis. Sci. Rep..

[37] Cantarel BL (2008). MAKER: An easy-to-use annotation pipeline designed for emerging model organism genomes. Genome Res.

[38] Parks DH, Imelfort M, Skennerton CT, Hugenholtz P, Tyson GW (2015). CheckM: Assessing the quality of microbial genomes recovered from isolates, single cells, and metagenomes. Genome Res..

[39] Seemann T (2014). Prokka: Rapid prokaryotic genome annotation. Bioinformatics.

[40] Le Han H, Nguyen TTH, Li Z, Shin NR, Kim SG (2022). *Cellulosimicrobium protaetiae* sp. Nov., isolated from the gut of the larva of *Protaetia brevitarsis seulensis*. Int. J. Syst. Evol. Microbiol..

[41] Ten LN (2006). *Paenibacillus panacisoli* sp. nov., a xylanolytic bacterium isolated from soil in a ginseng field in South Korea. Int. J. Syst. Evol. Microbiol..

[42] Sasser, M. (MIDI technical note 101. Newark, DE: MIDI inc, 1990).

[43] Liu Y, Le Han H, Zou Y, Kim S-G (2019). *Flavobacterium ustbae* sp. Nov., isolated from rhizosphere soil of Alhagi sparsifolia. Int. J. Syst. Evol. Microbiol..

[44] Komagata K, Suzuki KI (1987). Lipid and cell-wall analysis in bacterial systematics. Methods Microbiol..

[45] Minnikin D (1984). An integrated procedure for the extraction of bacterial isoprenoid quinones and polar lipids. J. Microbiol. Methods.

[46] Han HL (2022). *Halorubrum salinarum* sp. Nov., an extremely halophilic archaeon isolated from a saturated brine pond of a saltern. Int. J. Syst. Evol. Microbiol..

