# Supplementary material for: Isolation of Spirosoma foliorum sp. nov. from the fallen leaf of Acer palmatum by a novel cultivation technique
Source: Sci Rep. 2023 Sep 6;13:14684. doi: 10.1038/s41598-023-35108-5 (PMC10482864; doi:10.1038/s41598-023-35108-5)
Supplement: Supplementary file 1 — Supplementary Information. [file 41598_2023_35108_MOESM1_ESM.docx]

**Isolation of *Spirosoma foliorum* sp. nov. from the fallen leaf of *Acer palmatum* by a novel cultivation technique**

Ho Le Han ^1^, Dian Alfian Nurcahyanto ^2^, Neak Muhammad ^3,4^, Yong-Jae Lee ^3^, Tra T. H. Nguyen ^3,4^, Song-Gun Kim ^3,4, *^, Sook Sin Chan ^5^, Kuan Shiong Khoo ^6^, Kit Wayne Chew ^7^, Pau Loke Show ^8,9,10,11, *^, Thi Ngoc Thu Tran ^12^*,* Thi Dong Phuong Nguyen ^12, *^, Chen Yaw Chiu ^13, *^

^1^ The University of Danang, University of Science and Technology, 54 Nguyen Luong Bang St., Danang 550 000, Viet Nam.

^2^ Research Center for Biosystematics and Evolution, Research Organization for Life Sciences and Environment, National Research and Innovation Agency (BRIN), Indonesia.

^3^ Biological Resource Center/Korean Collection for Type Cultures (KCTC), Korea Research Institute of Bioscience and Biotechnology, 181 Ipsingil, Jeongeup, Jeonbuk 56212, Korea

^4^ University of Science and Technology (UST), 217 Gajeong-ro, Yuseong, Daejeon 34113, Korea

^5^ Institut Sains Biologi, Fakulti Sains, Universiti Malaya, Kuala Lumpur, Malaysia.

^6^ Department of Chemical Engineering and Materials Science, Yuan Ze University, Taoyuan, Taiwan.

^7^ School of Chemistry, Chemical Engineering and Biotechnology, Nanyang Technological University, 62 Nanyang Drive, Singapore, 637459 Singapore.

^8^ Department of Chemical Engineering, Khalifa University, Shakhbout Bin Sultan St - Zone 1 - Abu Dhabi - United Arab Emirates.

^9^ Zhejiang Provincial Key Laboratory for Subtropical Water Environment and Marine Biological Resources Protection, Wenzhou University, Wenzhou 325035, China.

^10^ Department of Sustainable Engineering, Saveetha School of Engineering, SIMATS, Chennai 602105, India.

^11^ Department of Chemical and Environmental Engineering, Faculty of Science and Engineering, University of Nottingham Malaysia, Jalan Broga, 43500 Semenyih, Selangor Darul Ehsan, Malaysia

^12^ The University of Da Nang, University of Technology and Education, Da Nang City 550000, Viet Nam.

^13^ Biochemical Engineering Research Center, Ming Chi University of Technology, New Taipei City 24301, Taiwan.

**Corresponding authors:**

**Professor. Dr. Chen Yaw Chiu** (chenyaw.chiu@gmail.com)

Biochemical Engineering Research Center, Ming Chi University of Technology, New Taipei City 24301, Taiwan.

**Dr. Thi Dong Phuong Nguyen** (ntdphuong@ute.udn.vn)

The University of Da Nang, University of Technology and Education, 48 Cao Thang st, 550000 Da Nang City, Viet Nam

**Professor. Song-Gun Kim** ([sgkim@kribb.re.kr](mailto:sgkim@kribb.re.kr))

Biological Resource Center/Korean Collection for Type Cultures (KCTC), Korea Research Institute of Bioscience and Biotechnology, 181 Ipsingil, Jeongeup, Jeonbuk 56212, Korea

University of Science and Technology (UST), 217 Gajeong-ro, Yuseong, Daejeon 34113, Korea

**Professor Ts. Ir. Dr. Pau Loke Show** ([pauloke.show@ku.ac.ae](mailto:pauloke.show@ku.ac.ae))

Department of Chemical Engineering, Khalifa University, Shakhbout Bin Sultan St - Zone 1 - Abu Dhabi - United Arab Emirates.

Zhejiang Provincial Key Laboratory for Subtropical Water Environment and Marine Biological Resources Protection, Wenzhou University, Wenzhou 325035, China.

Department of Sustainable Engineering, Saveetha School of Engineering, SIMATS, Chennai 602105, India.

Department of Chemical and Environmental Engineering, Faculty of Science and Engineering, University of Nottingham Malaysia, Jalan Broga, 43500 Semenyih, Selangor Darul Ehsan, Malaysia Department of Sustainable Engineering, Saveetha School of Engineering, SIMATS, Chennai, India 602105.

**Category: New Taxa**: *Bacteroidetes*

**Running Title**: *Spirosoma foliorum* sp. nov.

**Keywords**: Bacteroidetes; complete genome sequencing; Nanopore technology; gliding


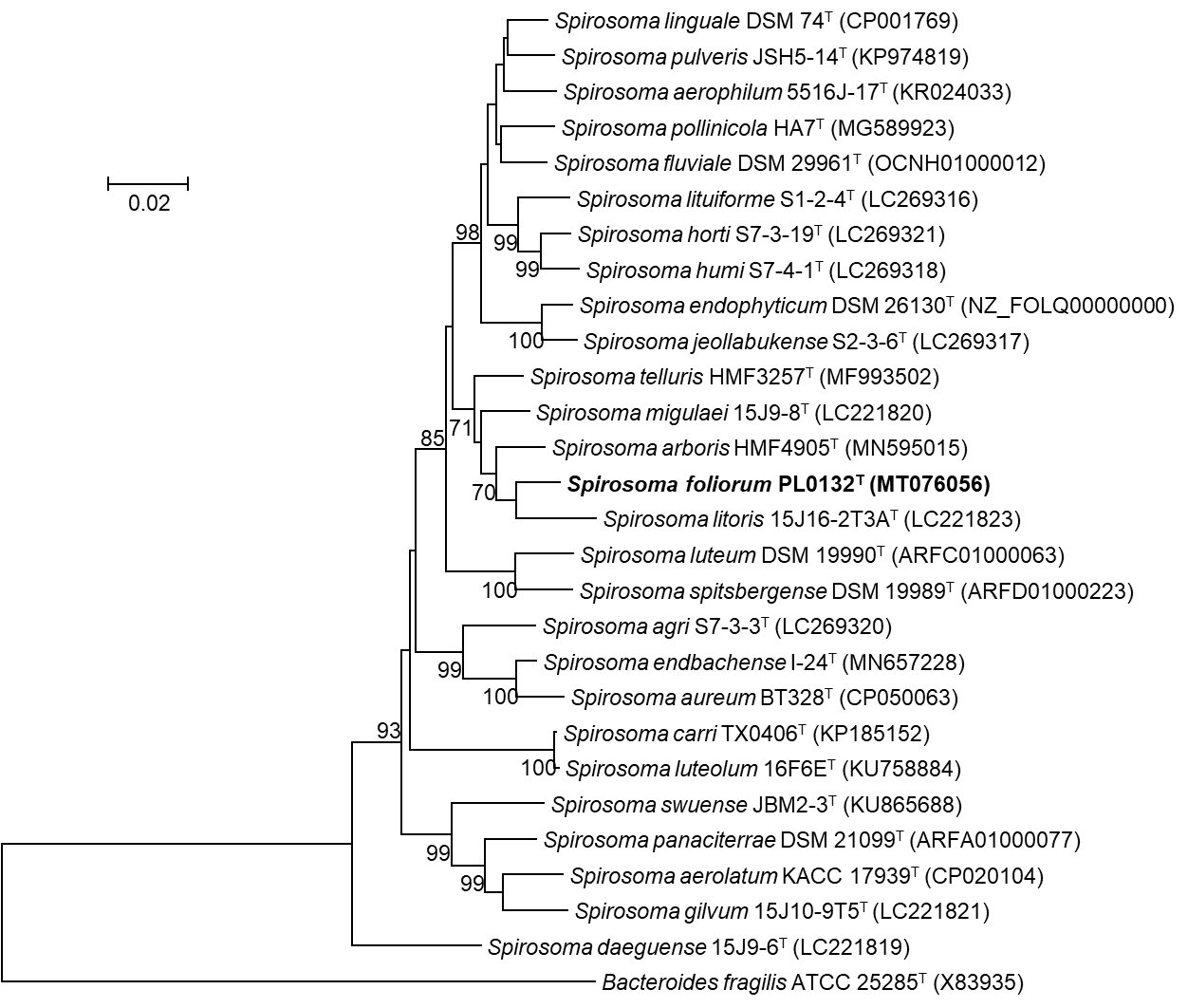


**Fig S1.** Neighbor-joining method tree based on the 16S rRNA gene sequences showing the phylogenetic location of strain *Spirosoma foliorum* PL0132^T^ with related taxa. The numbers at the nodes indicate the level of bootstrap values (>70 %) based on 1000 replications. Bar, 0.02 substitutions per nucleotide position.


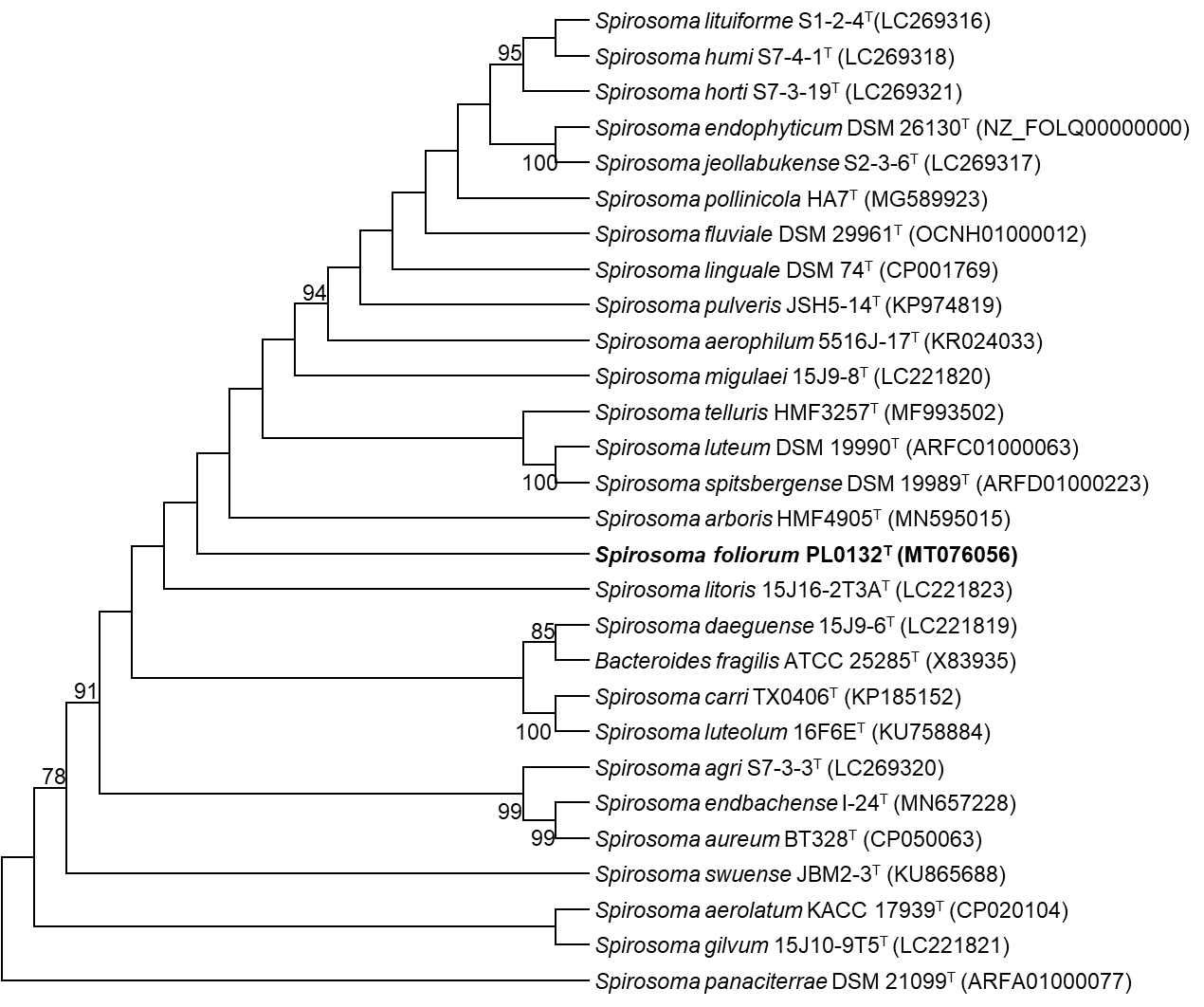


**Fig S2.**  Maximum-Parsimony phylogenetic tree, based on 16S rRNA gene sequences, showing the phylogenetic position of *Spirosoma foliorum* PL0132^T^ among related strains of the genus *Spirosoma* and representatives of other members of phylum *Bacteroidetes*. Bootstrap values (based on 1,000 replications) greater than 70% are shown at the branch points. The tree was rooted using *Bacteroides fragilis* ATCC 25285^T^ (X83935) as an outgroup.


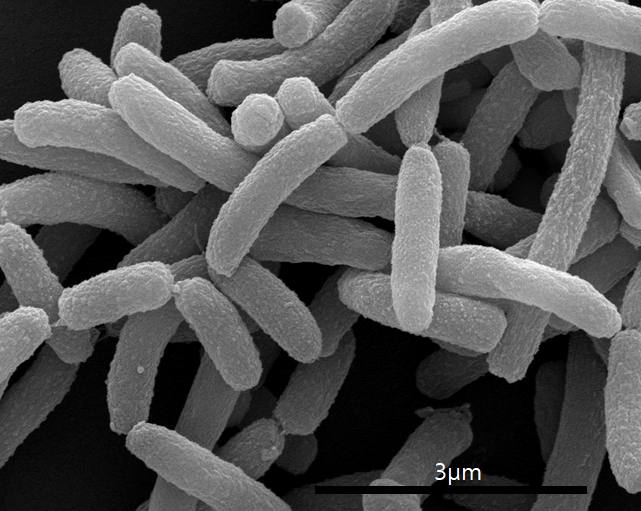


**Fig S3**. Scanning electron micrographs (SEM) of strain *Spirosoma foliorum* PL0132^T^ grown on R2A for 2 days at 25 ºC. Bars 3μm .


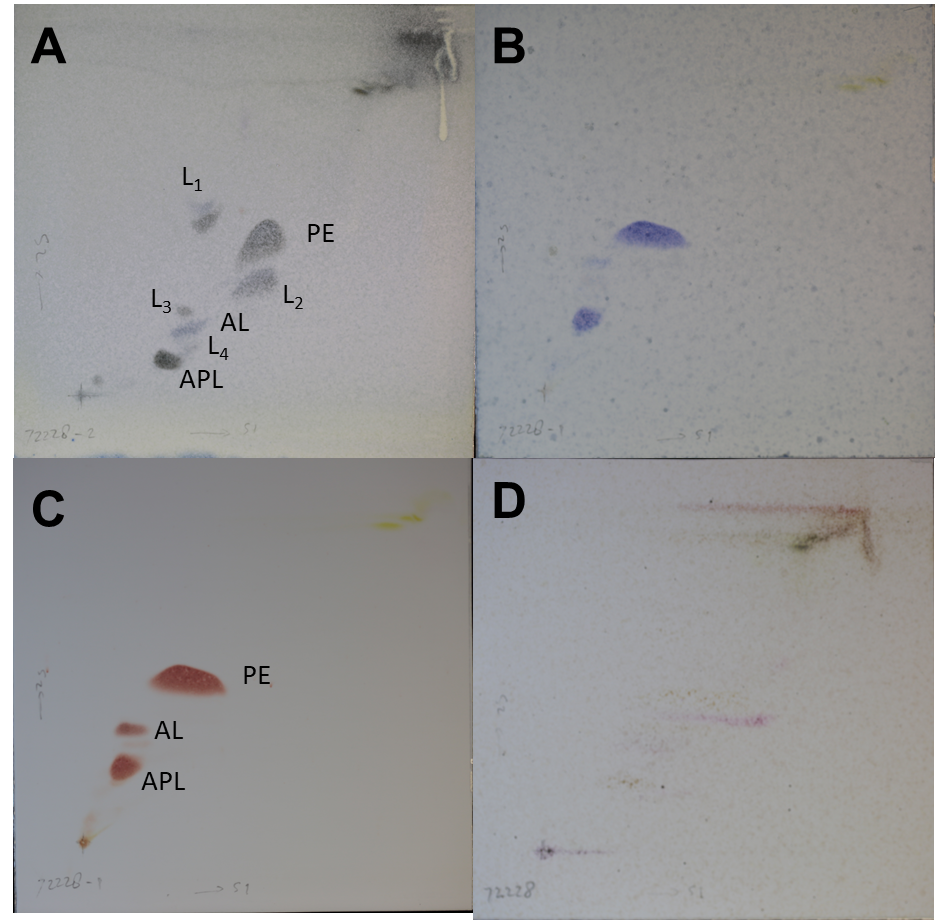


1^st^

2^nd^

**Fig S4.** Two-dimensional TLC patterns of the total polar lipids of strain *Spirosoma foliorum* PL0132^T^. The solvent systems used were chloroform-methanol-water (65: 25: 4, v/v) for the first dimension and chloroform-acetic acid-methanol-water (80: 18: 12: 4, v/v) for the second dimension. The following spray reagents were used for detection: A, molybdatophosphoric acid (for total lipids); B, molybdenum blue (for phospholipids); C, ninhydrin (for amino lipids) ;D α-naphthol (for glycolipids). Phosphatidylethanolamine (PE), Amino lipid (AL), Amino Phospho Lipid (APL), and unidentified lipids (L1-L4).1^st^ : first dimension; 2^nd^ : second dimension.


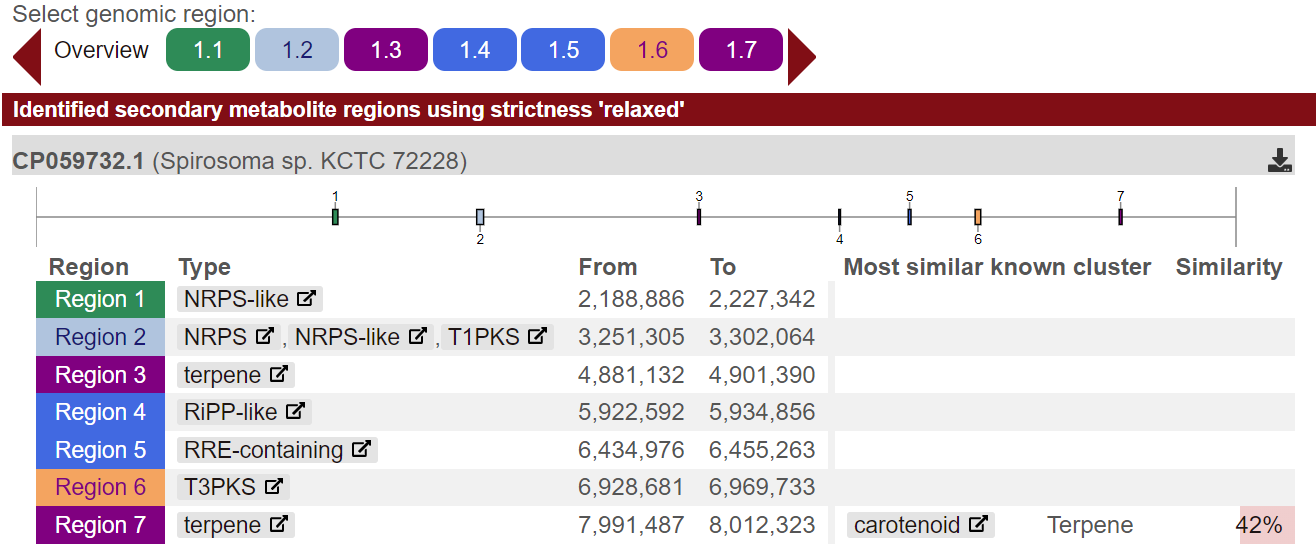


**Fig S5.** Predicted gene clusters of secondary metabolites biosynthesis annotated in antiSMASH against strain PL0132^T^ complete genome. Analyses provided the identification of six clusters involved in biosynthesis of terpene synthase genes, polyketide synthase type I and III (T1PKS and T3PKS), unspecified ribosomally synthesized and post-translationally modified peptide product cluster (RiPP-like), RRE-element containing cluster and non-ribosomal peptide synthetase (NRPS).
